# Gut microbiota and radiation-induced injury: mechanistic insights and microbial therapies

**DOI:** 10.1080/19490976.2025.2528429

**Published:** 2025-07-06

**Authors:** Lili Li, Zian Yang, Yuetao Yi, Yipeng Song, Wei Zhang

**Affiliations:** a Yantai Institute of Coastal Zone Research, Chinese Academy of Sciences, Yantai, China; b College of Marine Sciences, University of Chinese Academy of Sciences, Beijing, China; c Department of Radiotherapy, Yantai Yuhuangding Hospital, Yantai, China

**Keywords:** Gut microbiota, microbial metabolites, radiotherapy, novel strategies

## Abstract

Radiotherapy represents a crucial therapeutic modality in cancer treatment, yet its efficacy is frequently limited by radiation-induced toxicity. Growing evidence indicates that gut microbiota and their metabolites serve as key regulators of both radioprotective and radiosensitizing effects. This review systematically examines three fundamental regulatory mechanisms through which gut microbiota and its metabolites mitigate radiation-induced injury: (1) modulation of intestinal epithelial cell regeneration and tumor cell apoptosis via Wnt/β-catenin and PI3K/AKT/mTOR pathways; (2) immunomodulation via Toll-like receptor activation and NF-κB signaling; (3) oxidative stress management via Nrf2 signaling. We also evaluate various microbiota-targeted interventions, ranging from probiotics and prebiotics to fecal microbiota transplantation and emerging engineered microbial therapies, highlighting their potential in clinical radiotherapy. Finally, we emphasize current limitations and future research directions, underscoring the need to overcome existing challenges in microbiome analysis and therapeutic durability to fully realize the potential of precision radio-microbiome medicine, which may provide valuable references for developing personalized radiotherapy strategies based on gut microbiota and their metabolites.

## GRAPHICAL ABSTRACT

**Figure:**
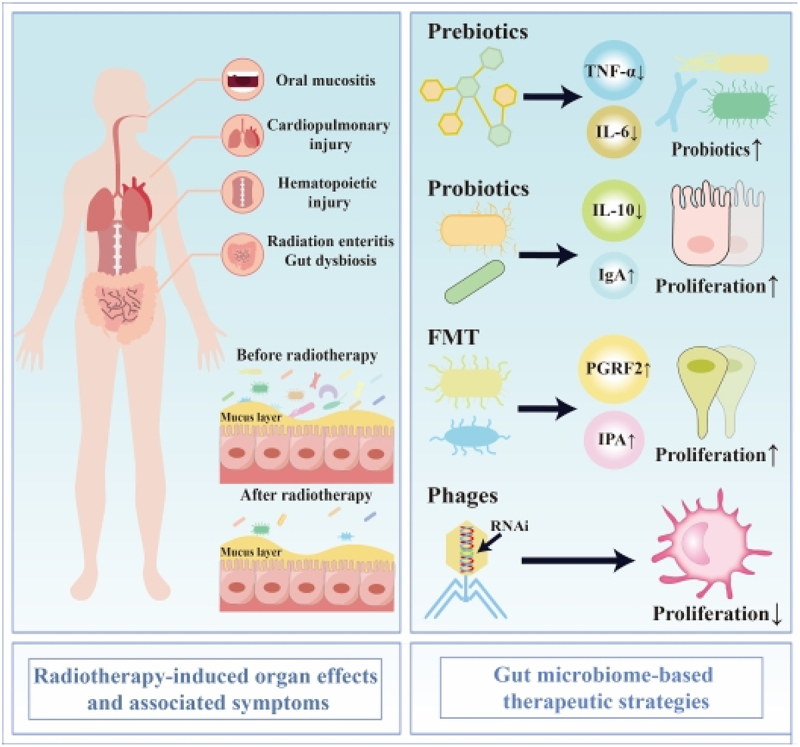

## 1. Introduction

Radiation protection is currently a critical global concern. With the development of nuclear energy and industrial incidents such as the discharge of nuclear wastewater into the ocean, ionizing radiation poses a significant threat to human health. There is an urgent need to develop effective radiation protection strategies. Radiation therapy is the most common source of radiation, widely used in medical diagnostics and cancer treatment. Research into the mechanisms of radiation-induced injury is essential for developing better radiation protection strategies. At present, over 50% of individuals diagnosed with cancer require radiotherapy as a common treatment. During the treatment process, various side effects or injuries often occur, including radiation-induced enteritis, mucositis, and hematological damage. These side effects not only seriously affect patients’ quality of life but also limit the clinical application and effectiveness of radiotherapy.^1,2^ To mitigate radiation-induced injury, various therapeutic approaches have been developed.^3^ In the past decade, there are increasing evidences on the role of gut microbiota and metabolites as key factors participating in important physiological and biochemical processes of radiation injury. Gut microbiota plays a critical role in the occurrence and development of radiation injury by regulating the host’s immune response, inflammatory pathways, and metabolic activities.^4^ Interventions based on the gut microbiome, such as fecal microbiota transplantation (FMT), supplementation with probiotics and prebiotics, and the application of bacterial metabolites, have been demonstrated to significantly alleviate radiation-induced injury.^5^ However, the existing methods mostly focused on symptom alleviation rather than addressing the mechanisms underlying the radiation-induced injury as the root causes, therefore limiting their preventive and therapeutic efficacy. This review aims to critically discuss the relationship between radiation-induced injuries and gut microbiota, as well as their microbial metabolites. It also highlights advances in understanding the molecular mechanisms underlying gut microbiota manipulation and in developing microbiota-related approaches to reduce radiotherapy-induced injury.

## 2. Gut microbiota dynamics in radiation injury

### 2.1. The factors and effects of radiation injury

Ionizing radiation induces complex pathological effects through both direct and indirect mechanisms. The primary damage stems from DNA strand breaks and water radiolysis-generated reactive oxygen species (ROS), which collectively disrupt cellular homeostasis. ROS overproduction induces oxidative damage to lipids, proteins, and nucleic acids and further compromises mitochondrial function by disrupting the electron transport chain. This leads to energy metabolism dysfunction and further ROS accumulation.^6^ In parallel, radiation-induced DNA damage activates cell cycle checkpoints and apoptotic pathways through ATM/ATR-p53 signaling.^7^ At the tissue level, these cellular injuries manifest as epithelial barrier dysfunction, mucosal erosion, and increased intestinal permeability. At the systemic level, radiation exposure initiates inflammatory cascades through the release of damage-associated molecular pattern (DAMP), which subsequently activate innate immune pathways including TLR and NLRP3 inflammasome signaling.^8^ Although these interconnected mechanisms underlie radiation toxicity, emerging evidence implicates gut microbiota in modulating these damage pathways. The following sections delineate the regulation of gut microbiota in radiation-induced injury.

### 2.2. Microbiota-mediated regulation of oxidative stress and DNA damage

Radiation-induced ROS and DNA damage are critically modulated by gut microbiota and their metabolic products. Accumulating evidence reveals that gut microbial metabolites critically regulate genomic stability and redox homeostasis. Specially, propionate treatment reduces phosphorylation of DNA damage markers (p53, 53BP1) and decreases ROS levels in intestinal and bone marrow stem cells of irradiated mice.^9^ Similarly, *Lactobacillus plantarum* enhance intestinal stem cell DNA repair capacity by activating the FXR-FGF15 axis, as evidenced by reduced the level of γ-H2AX (a biomarker of DNA double-strand breaks).^10^ Additionally, pretreatment with *Lactobacillus rhamnosus* GG (LGG) restores microbial composition, suppresses DNA damage, and attenuates inflammation by inhibiting the cGAS/STING pathway.^11^ These findings highlight the critical role of microbial metabolites in counteracting radiation-induced cellular damage.

### 2.3. Microbiota-mediated inflammation control and tissue repair

Radiation-induced DNA damage and ROS overproduction disrupt the intestinal epithelial barrier, creating an oxidative microenvironment that activates inflammatory pathways and disrupts the balance between pro- and anti-inflammatory factors. Consequently, gut microbiota critically influences both localized and distant organ inflammatory responses.

#### 2.3.1. Radiation-induced enteritis: microbial dysbiosis and barrier dysfunction

Radiation-induced enteritis is a common side effect of abdominal radiotherapy, characterized by dysbiosis of the gut microbiota and damage to the intestinal mucosa. It was demonstrated that the nuclear factor kappa B (NF-κB) signaling pathway was activated by intestinal pathogenic bacteria, such as *Enterobacteriaceae* and *Bacteroides* genera, which exacerbated enteritis during radiotherapy. Conversely, beneficial microbes, including *Faecalibacterium prausnitzii* and *Bifidobacterium infantis*, inhibit NF-κB activation, reducing the inflammatory response.^12^ Gut microbiota metabolites also play a significant role in the progression of enteritis. Studies have demonstrated that members of Lachnospiraceae family significantly ameliorate radiation enteritis by producing short-chain fatty acids (SCFAs).^9^ Furthermore, intestinal inflammation was characterized not only by increased inflammatory markers but also by compromised epithelial barrier integrity. Recent studies have revealed that in patients with enteritis, pathogenic bacteria such as *Citrobacter rodentium*, disrupt the mucus layer of the epithelial barrier, thereby exacerbating the condition. Conversely, beneficial bacterial species, including *Akkermansia muciniphila* and *Lactobacilli*, have been shown to promote mucus secretion and preserve the structural integrity of the intestinal epithelial barrier.^13^ Furthermore, studies have revealed that indole derivatives produced by the gut microbiota can reduce the production of inflammatory cytokines and maintain the integrity of the intestinal epithelium.^14^ These findings highlight the critical role of gut microbiota in the pathogenesis, progression, and therapeutic management of radiation-induced enteritis.

#### 2.3.2. Remote radiation effects: microbial metabolic regulation beyond the gut

Radiotherapy is also one of the crucial treatments for head and neck cancer. However, it may induce side effects such as oral mucositis. As the initial gateway of the digestive system, the oral microecological status influences the function and integrity of the intestinal barrier.^15^ Studies have shown that radiation-induced oral mucositis alters the oral microbiota. Among these, *Fusobacterium nucleatum* can invade and colonize the digestive tract, increasing the radiotherapy resistance of colorectal cancer and the severity of radiation-induced injury.^16^ However, oral administration of the probiotic cocktail significantly alleviates oral mucositis by enhancing patient immunity and modulating gut microbiome.^17^ In addition to radiation-induced oral mucositis, radiation-induced cardiopulmonary injury is another severe complication of thoracic radiotherapy.^18^ L-histidine derived from gut microbiota and their secondary metabolite, imidazole propionate (ImP), significantly improved lung respiratory and cardiac contractile functions in mice after radiation exposure.^19^ Hematopoietic injury is another common side effect of radiotherapy, due to the high concentration of radiosensitive proliferating cells in the bone marrow. The gut microbiota metabolite, Indole 3-propionic acid (IPA), increased the volume and weight of the thymus and spleen as well as the function of hematopoietic stem cells in mice.^20^ Propionate acid, another gut microbiota metabolite, alleviated DNA damage, promoted hematopoietic stem cell proliferation, and reduced reactive ROS generated in gastrointestinal tract tissue due to irradiation, thereby enhancing radioresistance in mice.^9^ In summary, these studies collectively demonstrate that gut microbial ecology plays an essential regulatory role in radiation-induced injury. Furthermore, we discuss the signaling pathways, receptors, and cytokines of the gut microbiota and its metabolites that regulate radiation-induced injury.

## 3. Mechanistic insights into microbial modulation of radiation-induced injury

**Figure 1.**
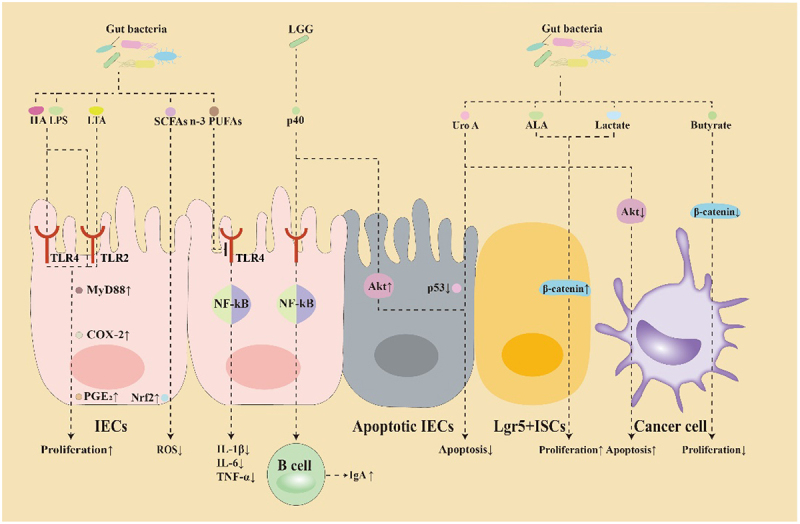
Mechanisms of gut microbiota and their metabolites in alleviating radiotherapy-induced symptoms.

The radiation fields required for malignant tumor treatment inevitably encompass substantial portions of the intestine, an organ that is highly susceptible to radiation toxicity. Over the past decade, extensive research has demonstrated that gut microbiota not only regulated tumor cell proliferation, metastasis and apoptosis, but also mitigated radiotherapy-induced adverse effects by modulating the key signaling pathways, inflammatory factors, and microbial metabolites.^21,22^ The following sections discuss the key mechanisms through which gut microbiota mediates radiation-induced intestinal injury (Figure 1).

The primary mechanisms involve three signaling pathways (Wnt, PI3K/AKT/mTOR, NF-κB), one transcription factor (Nrf2), and one receptor family (TLRs). hyaluronic acid (HA), lipopolysaccharide (LPS), and lipoteichoic acid (LTA) activate TLR4 and TLR2 on intestinal epithelial cells (IECs), promoting prostaglandin E2 (PGE2) secretion and enhancing IEC proliferation, reducing radiation-induced tissue damage. Short-chain fatty acids (SCFAs) activate the Nrf2 pathway in IECs, which mitigates the elevation of ROS induced by radiation. ω-3 polyunsaturated fatty acids (*n*-3 PUFAs) interact with TLR4 on IECs, suppressing the NF-κB pathway and reducing the levels of inflammatory cytokines (IL-1β, IL-6, TNF-α), thus alleviating radiation-induced inflammation. *Lactobacillus rhamnosus* GG-secreted p40 proteins activate epidermal growth factor receptor (EGFR) on IECs, stimulating the NF-κB pathway to upregulate expression of a proliferation-inducing ligand (APRIL), which promotes immunoglobulin A (IgA) production and facilitates intestinal barrier repair. Urolithin A (UroA) and the p40 proteins inhibit radiation-induced apoptosis in IECs by downregulating p53 and upregulating Akt expression, respectively. α-linolenic acid (ALA) and lactate activate the Wnt/β-catenin pathway, promoting the proliferation of Lgr5^+^ ISCs and differentiation, whereas reducing radiation-induced cellular damage. UroA and butyrate enhance tumor apoptosis and inhibit tumor proliferation by suppressing the PI3K/AKT/mTOR and Wnt/β-catenin pathways, respectively.

### 3.1. Radiation-induced side effects mediated by intercellular signaling

#### 3.1.1. Wnt/β-catenin signaling pathway – epithelial regeneration

The Wnt/β-catenin signaling pathway is a crucial therapeutic target in radiotherapy. Its inhibition disrupts tumor DNA repair and induces apoptosis, whereas its activation stimulates intestinal stem cell proliferation to protect against radiation-induced injury.^23^ Emerging evidence indicates that SCFAs produced by *Ruminococcus* species activate the Wnt signaling cascade through α-linolenic acid (ALA), subsequently promoting mouse intestinal stem cells (ISCs) proliferation and enhancing epithelial regeneration following radiation-induced injury.^24,25^ Furthermore, studies demonstrate that the gut microbiota-derived metabolite indole-3-carboxaldehyde (I3A), when administered via oral gavage, ameliorates radiation-induced intestinal injury in mice by activating the AhR/IL-10/Wnt signaling pathway to promote intestinal epithelial cell proliferation, thereby mitigating mucosal damage and maintaining intestinal barrier integrity.^26^ Additionally, lactate, a major microbial metabolite in gut, has been shown to stimulate Wnt/β-catenin signaling and promote the renewal of Lgr5^+^ ISCs, thereby providing protection against radiation-induced intestinal toxicity in mice undergoing radiotherapy.^27^ The results of clinical trials have indicated that sodium butyrate enemas are an effective treatment for radiation proctitis, reducing the frequency of bowel movements and pain experienced by patients, and significantly enhancing their quality of life.^28^ In summary, as a key mechanism of cellular response to radiotherapy, targeting and inhibiting Wnt/β-catenin signaling pathway represents a key mechanism for enhancing radiosensitivity and mitigating radiation-induced injury.

#### 3.1.2. PI3K/AKT/mTOR signaling pathway – cell survival regulation

The PI3K/Akt/mTOR signaling pathway is crucial for regulating essential cellular functions such as differentiation, proliferation, apoptosis, and DNA repair.^29^ Research has shown that abnormal activation and dysregulation of the PI3K/Akt/mTOR signaling pathway frequently occur in tumor cells, often resulting in uncontrolled tumor proliferation and impaired DNA damage repair.^30^ For example, studies have indicated that activation of the PI3K/Akt/mTOR pathway correlates with radiotherapy resistance in patients with head and neck squamous cell carcinoma (HNSCC). Inhibitors of this pathway are considered potential radiosensitizers.^31^ Furthermore, studies have demonstrated that feeding mice sitosterols significantly increased SCFAs secretion in gut and markedly decreased phosphorylation of protein kinase B (Akt). This cascade results in the release of cytochrome c from mitochondria into the cytosol, thereby elevating the levels of caspase-3 and caspase-9, enhancing poly ADP-ribose polymerase (PARP) cleavage, and inducing tumor cell apoptosis.^32^ Additionally, urolithin A (UroA), a ubiquitous microbial metabolite in the gut, has been demonstrated to impede tumor cell proliferation and induce apoptosis via the inhibition of this signaling pathway.^33^ In the context of immunosuppressive tumor microenvironments, UroA has been shown to reduce immunosuppressive components such as tumor-associated macrophages (TAMs), thereby enhancing the effects of radiotherapy. Additional studies indicated that UroA mitigated radiation-induced intestinal symptoms and provided radioprotection in mice by regulating the expression of the tumor suppressor p53, promoting Akt phosphorylation, and decreasing the relative abundance of *Bacteroidaceae*, *Enterobacteriaceae*, *Bifidobacteriaceae*, and *Erysipelotrichaceae* following radiation exposure.^34,35^

#### 3.1.3. NF-κB transcription factor signaling pathway – inflammatory control

NF-κB, a family of widely expressed transcription factors, plays a crucial role in regulating gene expression, particularly in the immune response, inflammation, cell growth, apoptosis, and tumorigenesis. Research has demonstrated that neoplastic cells often exhibit constitutive activation of NF-κB, leading to dysregulated cell cycle progression, uncontrolled proliferation, and resistance to apoptosis.^36^ Radiation-induced gut microbiota dysbiosis activates NF-κB, which subsequently triggering the production of a series of inflammatory cytokines.^37^ Omega-3 polyunsaturated fatty acids (PUFAs) derived from gut microbiota inhibited NF-κB activation through Toll-like receptor 4 (TLR4)/inhibitor of kappa B kinase (IKK) interaction, reducing inflammatory mediators (IL-1β, IL-6, TNF-α) in irradiated mice. Clinical studies demonstrated that omega-3 PUFAs promoted beneficial bacteria growth, reversed microbiota dysbiosis, and alleviated chemoradiotherapy-induced inflammation and oxidative stress.^38^ Furthermore, research indicated that gut microbiota following FMT secretes elevated levels of prostaglandin F2α (PGF2α), which mitigated radiation-induced injury in mice through the activation of the mitogen-activated protein kinase (MAPK)/NF-κB signaling pathway and suppresses inflammation factor production.^39^

### 3.2. Microbial pattern recognition: toll-like receptor activation

Toll-like receptors (TLRs) are crucial components of the immune system that significantly influence the host immune response by recognizing pathogen-associated molecular patterns (PAMPs) and DAMPs. Radiation functioned as a direct activator of TLRs, which were integral to the pathogenesis of radiation-induced injuries, inflammation, and cellular repair processes.^8^ Previous studies have demonstrated that Toll-like receptor 2 (TLR2) and TLR4, along with their respective ligands, played pivotal roles in mediating radioprotection through the myeloid differentiation primary response protein 88 (MyD88) pathway. Both TLRs mitigated the effects of radiation-induced apoptosis and excessive oxidative stress in the intestines of mice.^40,41^ Lipoteichoic acid (LTA) derived from LGG specifically engaged TLR2 on subepithelial macrophages, triggering MyD88-dependent signaling pathway. This TLR2 activation induced robust CXCL12 secretion, creating a chemotactic gradient that actively recruited mesenchymal stem cells (MSCs) to intestinal crypt niches. Recruited constitutive cyclooxygenase-2 (COX-2)-expressing MSCs produced prostaglandin E2 (PGE2), which protected epithelial stem cells against radiation-induced apoptosis in mice.^42,43^ In the context of TLR4, dendritic cells utilized TLR4/MyD88 to process antigens from dying tumor cells, enhancing antitumor immunity during radiotherapy. Previous studies have shown that lipopolysaccharide (LPS) and hyaluronic acid (HA) conferred radioprotection by activating TLR4 signaling and COX-2. In mice pretreated with LPS before radiotherapy, intestinal crypt cell survival increased significantly due to COX-2-mediated PGE2 production, promoting post-irradiation repair and proliferation of intestinal crypt cells following irradiation.^44,45^

### 3.3. Oxidative stress management: Nrf2 pathway

Nrf2 (nuclear factor erythroid 2-related factor 2) is a crucial transcription factor that plays a significant role in antioxidant defense, enhances DNA damage repair capacity, and consequently increases cellular resistance to radiotherapy.^46^ Previous studies have shown that 3,3’-diindolylmethane (DIM) significantly alleviated radiation-induced hematopoietic system injury and intestinal epithelial damage in irradiated mice. These protective effects are mediated through Nrf2-dependent upregulation of heme oxygenase-1 (HO-1), which promotes the regeneration of ISCs enriched with G-protein-coupled receptors.^47^ Interestingly, recent research indicated that acid-producing gut microbiota, including *Lactobacillus acidophilus*, facilitate the conversion of dietary indole-3-carbinol (I3C) into 2-(indol-3-ylmethyl)-3,3’-diindolylmethane (LTr1) and DIM, both of which are recognized anticancer agents. These findings, elucidated novel mechanisms through which gut microbiota ameliorated radiation-induced injury.^48^ Furthermore, hydrogen sulfide (H_2_S) produced by gut microbiota directly activated the Keap1/Nrf2/ARE cytoprotective signaling axis, leading to the upregulation of antioxidant proteins and glutathione. This mitigated oxidative injury to normal tissues and provided protection against the adverse effects associated with radiotherapy.^49,50^ In summary, gut microbiota and their metabolites serve to mitigate radiation-induced injury and enhance the efficacy of radiotherapy through the activation of multiple signaling pathways. Therapeutic strategies based on these mechanisms, harnessing the gut microbiota and its metabolites, have already been employed to alleviate radiation-induced injury. The following section introduces treatment strategies.

## 4. Microbiota-targeted interventions for radiation injury management

Conventional approaches to treating radiation-induced injury, including glucocorticoids, free radical scavengers and antibiotics, are often limited by adverse effects such as immunosuppression, single-target mechanism, adverse effects, metabolic dysregulation.^51^ Microbiota-based therapeutic strategies offer distinct advantages over conventional treatments for radiation-induced injury: (1) Multimodal Mechanisms: The diverse array of gut microbiota-derived metabolites, SCFAs, indole derivatives, and tryptophan metabolites, exert synergistic radioprotection through coordinated modulation of multiple signaling pathways; (2) Temporal Flexibility: These interventions can be administered either prophylactically before radiation exposure to prevent injury or therapeutically after irradiation to promote tissue recovery, offering adaptable treatment windows; (3) Reduced Toxicity: As naturally occurring commensal organisms, gut microbiota mediates biological regulation without introducing the toxic side effects associated with traditional drugs; and (4) Polypharmacological Effects: Unlike single-target therapies, microbiota-based approaches simultaneously address multiple pathological processes, including immunomodulation, epithelial barrier repair, and stem cell maintenance, resulting in more holistic therapeutic outcomes.^52^ We discuss the current gut microbiota-based therapeutic strategies for mitigate radiation-induced injury in the following sections.

### 4.1. Prebiotic supplementation strategies

Prebiotics are defined as non-digestible or poorly digestible food ingredients that selectively stimulate the growth and activity of beneficial bacteria within the human gastrointestinal tract. Our research has demonstrated that prebiotics, exemplified by dietary fiber, and their subsequent breakdown by gut microbiota into a range of micro- and macro-nutrients, including polyphenols and oligofructose (FOS), can regulate the composition of gut microbiota through various pathways, thereby influencing human health.^53,54^ Research on their application in radiotherapy has demonstrated that prebiotics can effectively alleviate symptoms such as gut dysbiosis and modulate inflammatory factors in patients following radiotherapy.^55^ Furthermore, studies have indicated that the supplementation of a mixture of inulin and fructooligosaccharides during radiation therapy resulted in the recovery of *Lactobacillus* and *Bifidobacterium* populations after the conclusion of treatment, as well as improved diarrhea in patients with gynecological cancer undergoing pelvic radiotherapy.^56^ Additionally, it has been reported that inulin can increase the relative abundance of fecal *Bacteroides acidifaciens*, which stimulated the production of SCFAs, thereby enhancing the intestinal microenvironment. The *Bacteroides acidifaciens* decelerated the proliferation of bladder cancer cells during radiation therapy and increased the radiosensitivity of bladder cancer cells.^57^ In a murine model of radiation-induced pulmonary fibrosis, phycocyanin administration resulted in a reduction in the abundance of inflammation-related bacteria and a decrease in the levels of inflammatory factors, including TNF-α, LPS, and IL-6. Furthermore, it diminished collagen fiber deposition in lung tissue, the intestinal tract, and serum.^58^ Similarly, studies have demonstrated that oral konjac glucomannan (KGM) significantly alleviated whole-body radiation-induced hematopoietic and intestinal injury in mice. Additionally, KGM inhibited radiation-induced apoptosis in human intestinal epithelial cells.^59^ We also found that marine-derived prebiotics such as alginate oligosaccharide and fucoidan were able to ameliorate a range of intestinal dysfunctions such as gut dysbiosis, oxidative stress, inflammatory stress and other intestinal injury in mice by promoting the production of metabolites such as SCFAs and tryptophan-related metabolites by the gut microbiota.^60,61^

### 4.2. Probiotic-based radioprotection

Probiotics are defined as live microorganisms that confer benefits to the host, primarily by improving the intestinal microbiome and enhancing immune function.^62^ The most studied genera, *Akkermansia* and *Lactobacillus*, along with postbiotic, were primarily discussed.

#### 4.2.1. Lactobacillus rhamnosus GG

**Figure 2.**
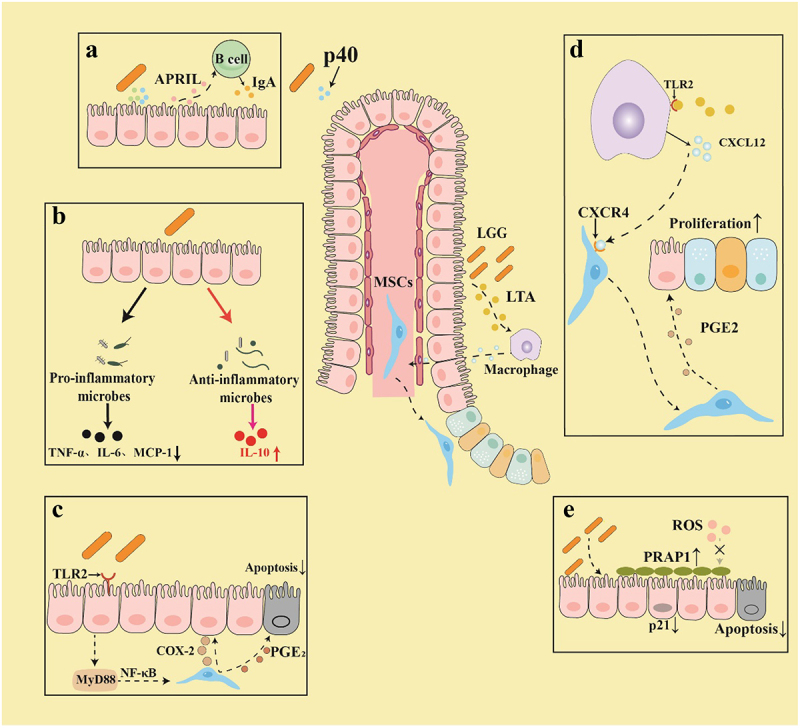
*Lactobacillus rhamnosus* GG (LGG) alleviates radiotherapy-induced injury through several mechanisms.

*Lactobacillus rhamnosus* has been extensively studied for its role in radiotherapy (Figure 2). It promotes the proliferation of epithelial cells to maintain intestinal barrier integrity, modulates intestinal immunity, and reshapes gut microbiota to mitigate radiation-induced injury.^11^ Pretreatment with *Lactobacillus rhamnosus* GG (the most effective strain) significantly reduces apoptosis in small intestinal epithelial cells and enhances crypt survival in mice. This effect is mediated by promoting the relocation of mesenchymal stem cells expressing COX-2 to the crypt base via the TLR2/MyD88 signaling pathway.^43^ Additional studies demonstrated that LGG released LTA, which bound to TLR2 on macrophages surrounding the crypts, inducing the migration of MSCs toward epithelial stem cells and promoting the production of PGE2, then protecting epithelial cells from radiation-induced apoptosis.^42^ Moreover, LGG supplementation increased the levels of intrinsically disordered proline-rich acidic protein 1 in the gastrointestinal epithelium of mice, which safeguarded intestinal cells from radiation-induced apoptosis and oxidative damage. This was achieved by forming a protective layer over the intestinal epithelial barrier and limiting the expression of p21.^63^

(a) LGG-secreted p40 proteins activate epidermal growth factor receptors (EGFR) on intestinal epithelial cells, promoting the production of the proliferation-inducing ligand (APRIL) ligand. This process ultimately induces B cells to produce IgA, ameliorating radiation-induced intestinal damage. (b) LGG inhibits the production of pro-inflammatory factors and promotes the production of anti-inflammatory factors. (c) The lipopolysaccharide (LPS) of LGG interacts with TLR2, promoting the production of MyD88, which activates the NF-κB signaling pathway. This activation stimulates mesenchymal stem cells (MSCs) to secrete cyclooxygenase-2 (COX-2), thereby protecting intestinal epithelial cells and facilitating the secretion of prostaglandin E2 (PGE2), which alleviates radiation-induced cell apoptosis. (d) LGG-secreted LTA interacts with TLR-2 on pericryptal macrophages, stimulating the release of the chemokine C-X-C motif chemokine ligand 12 (CXCL12). This promotes migration of MSCs toward the crypt base. Subsequently, these stem cells release PGE2, enhancing epithelial cell proliferation and mitigating radiation-induced crypt cells apoptosis. (e) LGG effectively promotes the expression of PRAP1 in the intestinal epithelium. PRAP1 is instrumental in maintaining the integrity of the epithelial barrier, protecting epithelial cells from oxidative damage by inhibiting p21 protein expression, which reduces cell apoptosis.

Furthermore, research has demonstrated that the p40 protein secreted by LGG effectively activated the epidermal growth factor receptor (EGFR) in intestinal epithelial cells and regulated the proliferation-inducing ligand (APRIL), thereby promoting the production of immunoglobulin A (IgA) in B cells and ultimately improving intestinal injury and inflammation.^64^ Moreover, LGG administration not only enriches anti-inflammatory commensals but also reduces the pro-inflammatory cytokine (MCP-1, IL-6, and TNF-α) in mice with radiation-induced enteritis.^65^ Furthermore, a probiotic mixture comprising *Lactobacillus rhamnosus* and *Lactobacillus acidophilus*has been demonstrated to restore the diversity and composition of gut microbiota in mice following whole-body irradiation at various doses, protects intestinal epithelial cells, promotes crypt proliferation, and prevents acute radiation-induced intestinal injury.^66^ Furthermore, a probiotic mixture containing LGG was found to enhance immunity, alleviate the severity of radiation-induced oral mucositis, and modulate gut microbiota associated with inflammatory responses during chemoradiotherapy in a clinical trial involving 99 patients with nasopharyngeal carcinoma.^17^

#### 4.2.2. Akkermansia muciniphila

**Figure 3.**
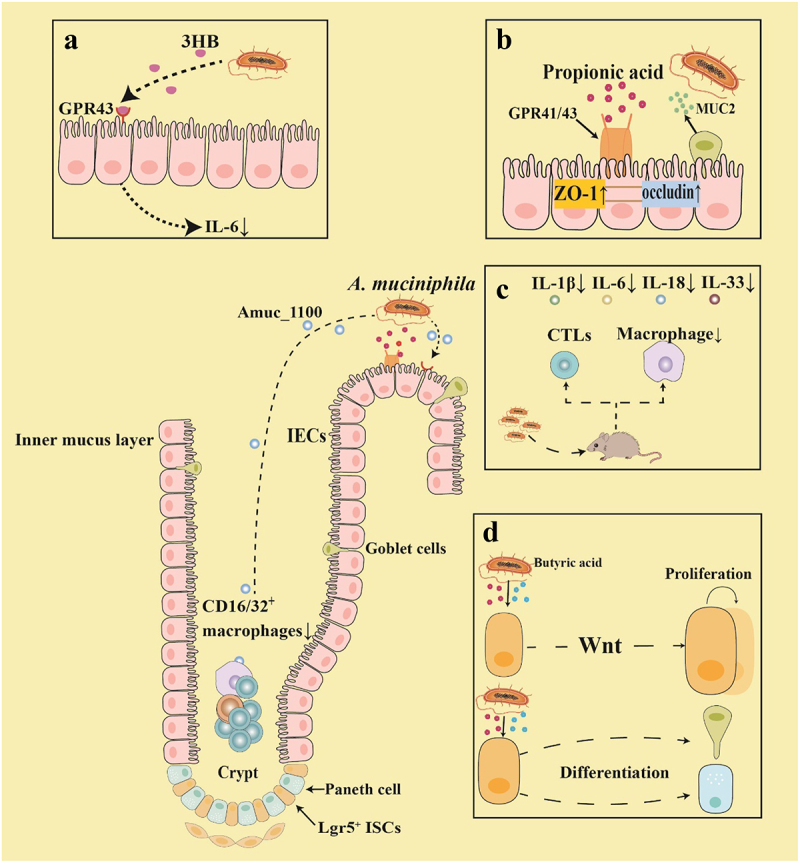
*A. muciniphila* alleviates radiotherapy-induced damage through several mechanisms.

*A. muciniphila* has garnered significant attention as a candidate for next-generation probiotics due to its effectiveness in enhancing gastrointestinal immunity (Figure 3), maintaining intestinal barrier integrity, regulating metabolism, and alleviating inflammatory diseases and complications related to chemotherapy and radiotherapy.^67^ Studies have demonstrated that *A. muciniphila* supplementation via gavage prior to radiotherapy significantly increased 30-day survival rates in irradiated mice.^68^ *A. muciniphila* significantly reduced macrophage infiltration and alleviated intestinal inflammation in irradiated mice by stably colonizing the gastrointestinal tract, promoting the proliferation of probiotic *Lactobacillus murinus*, and reshaping the gene expression profile of *Lactobacillus murinus*. ^69^ Oral administration of *A. muciniphila* significantly increased colon length, crypt density, and goblet cell numbers, whereas reduced levels of inflammatory markers (IL-6, TNF-α, and IL-1β) in the small intestine of irradiated mice.^70^ Furthermore, supplementation of *A. muciniphila* significantly enhanced intestinal biosynthesis of 3-hydroxybutyrate (3HB) in irradiated mice, which subsequently downregulated GPR43 receptor expression and attenuated IL-6 production, thereby ameliorating radiation-induced intestinal inflammatory injury.^71^ Another study demonstrated the therapeutic efficacy of inactivated *A. muciniphila* and its outer membrane protein Amuc_1100 in mitigating colonic inflammation through the immunomodulation of CD16/32^+^ macrophages and CD8^+^ cytotoxic T lymphocytes.^72^

(a) *A. muciniphila* promotes the synthesis of 3-hydroxybutyrate (3HB) in the intestine, which blocks G protein-coupled receptors 43 (GPR43)-mediated IL-6 signaling, thereby inhibiting the occurrence of radiation-induced enteritis. (b) Propionate secreted by *A. muciniphila* binds to GPR43, promoting the expression of tight junction membrane-integrated proteins and ZO-1 in intestinal epithelial cells. This action increases Mucin 2 (MUC2) levels in the epithelial barrier, thereby maintaining barrier integrity and mitigating radiation-induced intestinal damage. (c) Oral administration of *A. muciniphila* attenuates radiation-induced intestinal inflammatory response in mice by inhibiting pro-inflammatory cytokines (IL-1β, IL-6, IL-18, IL-33) and reducing cytotoxic T lymphocytes (CTLs) and macrophage infiltration. (d) *A. muciniphila* increased the concentration of SCFAs, which activated the Wnt signaling pathway, promoted ISC-mediated epithelial development, enhanced the proliferation of Lgr5^+^ ISCs and stimulated the differentiation of goblet cells and Paneth cells

*A. muciniphila* has been observed to mediate radioprotective effects primarily through production of propionic acid. Propionic acid is bound to GPR43 on the intestinal tract surface, promoting histone acetylation and thereby increasing the expression of tight junction proteins occludin and Zonula occludens-1. These proteins enhanced the structural integrity of the mucus layer on the surface of intestinal villi and concomitantly strengthened the intestinal epithelial barrier.^73,74^ Furthermore, pretreatment with *A. muciniphila* significantly attenuated combined radiation and methotrexate-induced intestinal injury in mice. Mechanistically, this protection was achieved through elevated SCFAs production that activated Wnt/β-catenin signaling pathway. The activation of signaling pathway enhances both the proliferation of Lgr5^+^ ISCs and the differentiation of goblet and Paneth cells, thereby mitigating radiation-induced injury.^75^ Therefore, *A. muciniphila* and LGG demonstrated significant potential in regulating intestinal health, enhancing intestinal immune homeostasis, and protecting against radiation injuries. These findings provide a crucial theoretical foundation for the development and application of *A. muciniphila* and LGG as novel types of strategies in the treatment of radiation-induced intestinal injury.

#### 4.2.3. Postbiotic and microbial metabolite approaches

In addition to the foregoing typical bacteria, other bacterial species have also been identified as playing a significant role in protecting against radiation-induced injuries. For example, *Lactobacillus rhamnosus* ATCC 7469 produces an extracellular polysaccharide that significantly mitigates oxidative and inflammatory stress induced by 1,2-dimethylhydrazine (DMH), even at low levels of ionizing radiation. This protective effect is mediated through the regulation of MAPK, phospho-signal transducer and activator of transcription 3 (STAT3), and β-catenin. Furthermore, this polysaccharide has been shown to improve the structural of integrity of DMH-induced colorectal cancer tissues, restore the functionality of the mucosal lining and goblet cells, and modulate the transcription of NF-κB and COX-2, thereby effectively controlling cancer progression.^76^

Furthermore, heat-killed *Salmonella typhimurium* has been demonstrated to alleviate lung congestion and pathological damage following irradiation by regulating transforming growth factor-β (TGF-β). Studies have shown that it reduced radiation-induced collagen deposition, prevented epithelial-mesenchymal transition (EMT), and inhibited apoptosis in lung tissue cells. Additionally, it significantly suppressed inflammatory cytokines such as IL-6, PGE2, TNF-α, contributing to the mitigation of radiation-induced lung injury.^77^ In another study, the cell-free supernatant of *Bacillus subtilis* significantly enhanced the activity of normal epithelial cells and increased the radiosensitivity of colorectal cancer cells by upregulating the expression of proapoptotic genes encoding bax and caspase-3.^78^ Furthermore, heat-killed *Lactobacillus acidophilus* significantly improved irradiation-induced intestinal injury by promoting the differentiation of intestinal epithelial cells (IECs) and the production of mucin-producing cells in the distal small intestine.^79^ Collectively, these studies underscore the potential of probiotics and their derivatives in preventing and treating oxidative stress, inflammation, and cancer, as well as their role in alleviating radiation-induced injury. Further investigation into these mechanisms will be essential for the development of therapeutic strategies.

### 4.3. Microbial ecosystem transplantation

FMT has emerged as a promising intervention for alleviating radiation-induced injuries. Microbiota transplantation primarily encompasses FMT and selective microbiota transplantation.^80^ Previous studies have demonstrated that FMT significantly enhances the survival rate and body weight of both male and female mice following total body irradiation. Additionally, it improved gastrointestinal function and epithelial integrity post-irradiation, providing protection against radiation-induced mortality. Notably, FMT considerably reduced the levels of FITC-dextran in the peripheral blood in response to elevated radiation, thickened the mucus layer, and significantly increased the number of goblet cells.^14^ Preliminary clinical trials involving approximately five patients have indicated that FMT effectively ameliorates intestinal symptoms and mucosal injury, including rectal bleeding, abdominal and rectal pain, diarrhea, and fecal incontinence. Three out of five patients experienced recovery from mucosal injury over time due to radiation enteritis.^81^ In the context of radiation-induced lung injury, FMT has been shown to improve lung function in mouse models, alleviate inflammatory responses, mitigate oxidative stress, and inhibit lung cell apoptosis by elevating the levels of gut microbiota-derived PGF2α.^39^ FMT also effectively increased the levels of gut microbiota-derived L-histidine, which promoted the production of ImP, a secondary metabolite of L-histidine. ImP significantly inhibited the NF-κB signaling pathway after irradiation and suppressed pyroptosis, thereby promoting pneumocyte proliferation. This process alleviated radiation-induced cardiopulmonary injury and improved heart systolic function and lung respiration.^19^ Furthermore, FMT effectively elevated the levels of microbe-derived IPA in the feces of irradiated mice, leading to a reduction in hematopoietic system injury and gastrointestinal toxicity.^82^ These studies suggest that the potential of FMT in treating radiation-induced injury warrants further exploration.

### 4.4. Emerging adjunctive therapies: phage, nutritional, and engineered microbial approaches

Additionally, emerging microbial therapies like phage therapy and nutritional intervention also show promise in enhancing microbiota-targeted radioprotective effects and treatment efficacy. Phage therapy involves the treatment of pathogenic bacterial infections through the lysis of bacteria by bacteriophages. In recent years, phages have been utilized as adjuncts to radiation therapy.^83^ For instance, bacteriophage Qβ particles have shown to inactivate NF-κB signaling, inhibit post-radiotherapy DNA repair, and reduce the growth rate of tumor cells by downregulating the expression of EGFR and IκB kinase in glioma cell lines. Furthermore, this approach enhanced the effectiveness of radiotherapy on glioblastomas in mice by enhancing cell penetration efficiency via convection-enhanced delivery.^84^ Additionally, research showed that RGD-targeted adeno-associated virus phages (RGD-AAVP-TNF) in combination with low-fraction radiotherapy synergistically modulates the immune system, leading to an increase in TAMs. This modulation subsequently controls the growth rate of primary syngeneic B16-F10 melanoma in female mice and prolongs their survival.^85^

Studies have shown that specific dietary components can indirectly enhance gut microbiota’s ability to mitigate adverse effects to radiotherapy and improve its efficacy. For instance, vitamin D3 supplementation promoted the expression of vitamin D receptors in the intestinal epithelium, leading to increased production of butyrate. This process significantly reduced opportunistic pathogens such as *Pseudomonas*, *Escherichia*, and *Shigella*, whereas increased populations of beneficial bacteria like *Lactobacillus* and *A. muciniphila*. Such changes contribute to the maintenance of gut microbiome homeostasis and enhance the effectiveness of radiotherapy.^86^ Furthermore, quercetin inclusion complex gels alter the composition of the gut microbiota in irradiated mice, resulting in a reduction in the abundance of *Firmicutes* and an increase in *Bacteroidota*. These gels also lower levels of IL-6 and TNF-α, reverse intestinal injury, provide significant neuroprotective effects, and reduce radiation-induced brain injury by modulating gut microbiota through the microbiota-gut-brain axis.^87^ We designed a novel compound, XH-105, based on the antioxidant activity of quercetin, which significantly reduces apoptosis in small intestinal villi and crypt cells of radiation-irradiated mice. This is achieved through the inhibition of the p53 signaling pathway, while promoting crypt proliferation and differentiation.^88^ In *vivo* studies demonstrate that ergothioneine hyaluronic acid gel acts as a radioprotectant, preventing radiation-induced gastroenteritis by reducing DNA damage and producing ROS production while inhibiting cell apoptosis, and reducing neutrophil infiltration in the gastrointestinal tract, thereby alleviating gastrointestinal inflammation.^89^

Genetically engineered gut microbiota plays a crucial role in the efficacy of radiotherapy. For instance, the engineered tumor-targeting probiotic *Escherichia coli* Nissle 1917 significantly promotes the secretion of catalase within the tumor microenvironment, catalyzing the conversion of H_2_O_2_ produced by tumor cells into O_2_, and enhancing ROS production under hypoxic conditions. This process alleviates tumor hypoxia and achieves radiosensitization in mice.^90^ Additionally, a study on the combination of *Bifidobacterium infantis* with radiotherapy in a murine lung cancer model demonstrated significant therapeutic benefits. The combination of *B. infantis* with monoclonal antibody therapy significantly reduces the expression levels of tumor biomarkers, including hypoxia-inducible factor-1α (HIF-1α) and TNF-α. This combination therapy increases apoptosis, decreased tumor cell proliferation, and alleviation of tumor hypoxia.^91^

## 5. Conclusions and future perspective

Gut microbiota has emerged as a pivotal determinant of radiotherapy outcomes, demonstrating dual roles in both radioprotection and radiosensitization. Although considerable progress has been achieved in characterizing microbial associations with radiation responses, critical knowledge gaps remain in three key aspects. First, the field remains dominated by correlative studies, with insufficient exploration of causality and incomplete analysis of related signaling pathways. For example, although Lachnospiraceae have been shown to ameliorate radiotherapy injury through tryptophan metabolism, the exact molecular mechanisms remain to be elucidated.^9^ Second, current gut microbiome analytical methodologies face two major technical limitations: (1) inherent methodological challenges including contamination risks, PCR amplification biases, low microbial biomass issues, and data redundancy, and (2) inadequate characterization of functionally important microbiome components (fungi, archaea, and viruses). These understudied microorganisms constitute critical mediators in elucidating the causal relationship between gut microbes and radiotherapy response.^92,93^ Third, clinical translation faces practical challenges. Most studies rely on fecal microbiome as a proxy, which inadequately represents region-specific microbial heterogeneity within the gastrointestinal tract.^94^ The limited gut microbiota diversity in current studies restrict the efficacy of microbiome-based radioprotective agents, since radiation dose and target tissue heterogeneity elicit divergent biological responses. In terms of clinical applications, existing microbial therapies often show low colonization rates and unsustainable efficacy, which mainly stems from the difficulty of exogenous microorganisms to stably colonize the gut for a long period of time.^95^

To advance this field, future research should focus on the development of cost-effective and high-resolution next-generation sequencing technologies, coupled with AI-driven bioinformatics analyzes to improve the identification of functional microorganisms. Concurrently the integration of multi-omics and machine-learning systems will be critical for elucidating the molecular mechanisms through which gut microbiota influence radiotherapy responses. On this basis, develop ingestible sampling sensors (smart capsules) to collect microbial samples from specific intestinal regions, construct a whole gut microbial profile, and screen radiotherapy-related biomarkers to establish a flora-marker-radiotherapy response association network.^96^ In addition, real-time monitoring of gut microbiota dynamics by using wearable devices with gut microarrays will help to guide the personalized modulation of probiotics, prebiotics, and postbiotics, to promote stable colonization of beneficial bacterial strains and ultimately achieve precision radiotherapy. These breakthroughs will push the application of gut microbiome in tumor radiotherapy from empirical intervention to the era of precision medicine.
